# 1-(2-Methoxy­anilino)anthraquinone

**DOI:** 10.1107/S1600536810013310

**Published:** 2010-04-21

**Authors:** Lihua Lu, Liang He

**Affiliations:** a College of Chemical & Pharmaceutical, Qingdao Agriculture University, Qingdao 266109, People’s Republic of China; b State Key Laboratory of Fine Chemicals, Dalian University of Technology, Dalian 116012, People’s Republic of China

## Abstract

In the title compound, C_21_H_15_NO_3_, the dihedral angle formed between the aromatic ring systems is 71.50 (3)°. The meth­oxy group is coplanar with the benzene ring to which it is connected, the C—O—C—C torsion angle being 6.37 (17)°. The observed conformation is stabilized by an intra­molecular N—H⋯O hydrogen bond, generating an *S*(6) ring.

## Related literature

For background to anthraquinone derivatives, see: Matsui (1998 ▶); Rao & Choudhary (1990 ▶).
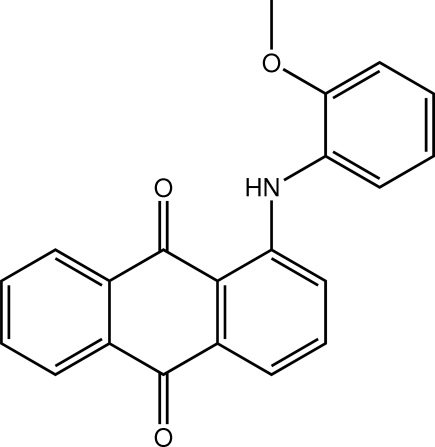


## Experimental

### 

#### Crystal data

C_21_H_15_NO_3_
*M* *_r_* = 329.34
Monoclinic, 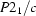

*a* = 11.4222 (3) Å
*b* = 7.9878 (2) Å
*c* = 16.9332 (4) Å
β = 100.9851 (12)°
*V* = 1516.65 (7) Å^3^
*Z* = 4
Mo *K*α radiation
μ = 0.10 mm^−1^
*T* = 110 K
0.40 × 0.30 × 0.14 mm

#### Data collection

Bruker–Nonius X8 APEXII diffractometer
Absorption correction: multi-scan (*SADABS*; Bruker, 2006 ▶) *T*_min_ = 0.962, *T*_max_ = 0.987
32405 measured reflections
5620 independent reflections
3863 reflections with *I* > 2σ(*I*)
*R*_int_ = 0.031

#### Refinement

*R*[*F* ^2^ > 2σ(*F* ^2^)] = 0.052
*wR*(*F* ^2^) = 0.161
*S* = 1.05
5620 reflections
286 parameters
All H-atom parameters refined
Δρ_max_ = 0.49 e Å^−3^
Δρ_min_ = −0.20 e Å^−3^

### 

Data collection: *APEX2* (Bruker, 2006 ▶); cell refinement: *SAINT* (Bruker, 2006 ▶); data reduction: *SAINT*; program(s) used to solve structure: *SIR92* (Altomare *et al.*, 1994 ▶); program(s) used to refine structure: *XL* (Bruker, 2004 ▶); molecular graphics: *XL*; software used to prepare material for publication: *XL*.

## Supplementary Material

Crystal structure: contains datablocks I, global. DOI: 10.1107/S1600536810013310/tk2648sup1.cif

Structure factors: contains datablocks I. DOI: 10.1107/S1600536810013310/tk2648Isup2.hkl

Additional supplementary materials:  crystallographic information; 3D view; checkCIF report

## Figures and Tables

**Table 1:** Hydrogen-bond geometry (Å, °)

*D*—H⋯*A*	*D*—H	H⋯*A*	*D*⋯*A*	*D*—H⋯*A*
N1—H1⋯O1	0.932 (19)	1.886 (18)	2.6279 (13)	135.0 (17)

**Fig. 1.**
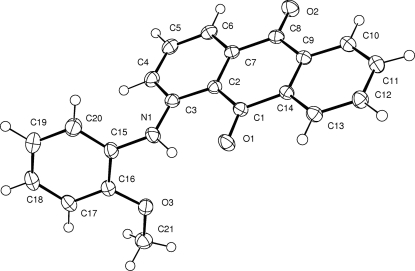
The molecular structure of (I) showing the atom-labelling scheme and displacement ellipsoids at the 50% probability level.

## supplementary crystallographic information

### Comment

Anthraquinone and its derivatives are important compounds in the fields of dyes and pigments (Matsui 1998; Rao & Choudhary, 1990). Here, we report the crystal structure of the title compound, (I), Fig. 1. The dihedral angle formed between the aromatic systems is 71.50 (3)°. The methoxy group is co-planar with the benzene ring to which it is connected with the C21–O3–C16–C17 torsion angle being 6.37 (17) °. The observed conformation is stabilised by an intramolecular N–H···O hydrogen bond, Table 1.

### Experimental

The crystal was obtained by dissolving (I) in methyl acetate (50 ml) and leaving the resulting solution, which was covered with Parafilm plastic containing pin holes, to evaporate at room temperature.

### Refinement

The hydrogen atoms were refined freely: N–H = 0.932 (19) Å, and C–H = 0.937 (16)–1.051 (17) Å.

### Crystal data

**Table:**

C_21_H_15_NO_3_	*F*(000) = 688
*M**_r_* = 329.34	*D*_x_ = 1.442 Mg m^−^^3^
Monoclinic, *P*2_1_/*c*	Mo *K*α radiation, λ = 0.71070 Å
Hall symbol: -P 2ybc	Cell parameters from 8857 reflections
*a* = 11.4222 (3) Å	θ = 2.8–31.7°
*b* = 7.9878 (2) Å	µ = 0.10 mm^−^^1^
*c* = 16.9332 (4) Å	*T* = 110 K
β = 100.9851 (12)°	Prism, red
*V* = 1516.65 (7) Å^3^	0.40 × 0.30 × 0.14 mm
*Z* = 4	

### Data collection

**Table:**

Bruker–Nonius X8 APEXII diffractometer	5620 independent reflections
Radiation source: fine-focus sealed tube	3863 reflections with *I* > 2σ(*I*)
graphite	*R*_int_ = 0.031
φ scans	θ_max_ = 34.9°, θ_min_ = 2.8°
Absorption correction: multi-scan (*SADABS*; Bruker, 2006)	*h* = −17→16
*T*_min_ = 0.962, *T*_max_ = 0.987	*k* = −12→11
32405 measured reflections	*l* = −24→25

### Refinement

**Table:**

Refinement on *F*^2^	Primary atom site location: structure-invariant direct methods
Least-squares matrix: full	Secondary atom site location: difference Fourier map
*R*[*F*^2^ > 2σ(*F*^2^)] = 0.052	Hydrogen site location: inferred from neighbouring sites
*wR*(*F*^2^) = 0.161	All H-atom parameters refined
*S* = 1.05	*w* = 1/[σ^2^(*F*_o_^2^) + (0.0934*P*)^2^ + 0.1215*P*] where *P* = (*F*_o_^2^ + 2*F*_c_^2^)/3
5620 reflections	(Δ/σ)_max_ = 0.001
286 parameters	Δρ_max_ = 0.49 e Å^−^^3^
0 restraints	Δρ_min_ = −0.20 e Å^−^^3^

### Special details

**Table:**

Geometry. All esds (except the esd in the dihedral angle between two l.s. planes) are estimated using the full covariance matrix. The cell esds are taken into account individually in the estimation of esds in distances, angles and torsion angles; correlations between esds in cell parameters are only used when they are defined by crystal symmetry. An approximate (isotropic) treatment of cell esds is used for estimating esds involving l.s. planes.
Refinement. Refinement of *F*^2^ against ALL reflections. The weighted *R*-factor wR and goodness of fit *S* are based on *F*^2^, conventional *R*-factors *R* are based on *F*, with *F* set to zero for negative *F*^2^. The threshold expression of *F*^2^ > σ(*F*^2^) is used only for calculating *R*-factors(gt) etc. and is not relevant to the choice of reflections for refinement. *R*-factors based on *F*^2^ are statistically about twice as large as those based on *F*, and *R*- factors based on ALL data will be even larger.

### Fractional atomic coordinates and isotropic or equivalent isotropic displacement parameters (Å^2^)

**Table:**

	*x*	*y*	*z*	*U*_iso_*/*U*_eq_	
O1	0.13993 (7)	0.20278 (10)	0.63675 (5)	0.02712 (19)	
O2	−0.22023 (8)	−0.23872 (11)	0.55875 (5)	0.0302 (2)	
O3	0.46774 (7)	−0.06574 (11)	0.66601 (4)	0.0286 (2)	
N1	0.26972 (8)	−0.02097 (13)	0.72798 (6)	0.0258 (2)	
H1	0.2651 (17)	0.076 (2)	0.6971 (11)	0.058 (5)*	
C1	0.05822 (9)	0.09907 (14)	0.62187 (6)	0.0203 (2)	
C2	0.06875 (9)	−0.07113 (13)	0.65401 (6)	0.0192 (2)	
C3	0.17497 (9)	−0.12548 (14)	0.70664 (6)	0.0216 (2)	
C4	0.18047 (11)	−0.29225 (15)	0.73478 (6)	0.0263 (2)	
H4	0.2586 (12)	−0.3278 (18)	0.7699 (8)	0.030 (4)*	
C5	0.08556 (11)	−0.39923 (16)	0.71402 (7)	0.0279 (3)	
H5	0.0911 (14)	−0.524 (2)	0.7336 (10)	0.043 (4)*	
C6	−0.02007 (11)	−0.34632 (15)	0.66463 (6)	0.0248 (2)	
H6	−0.0882 (13)	−0.4234 (19)	0.6527 (9)	0.034 (4)*	
C7	−0.02689 (9)	−0.18511 (14)	0.63397 (6)	0.0201 (2)	
C8	−0.13910 (9)	−0.13774 (14)	0.57762 (6)	0.0214 (2)	
C9	−0.14902 (9)	0.03492 (14)	0.54568 (6)	0.0198 (2)	
C10	−0.25212 (10)	0.08461 (15)	0.49214 (6)	0.0232 (2)	
H10	−0.3179 (11)	−0.0046 (17)	0.4743 (7)	0.020 (3)*	
C11	−0.26221 (10)	0.24721 (15)	0.46310 (6)	0.0249 (2)	
H11	−0.3326 (12)	0.2845 (18)	0.4248 (8)	0.029 (3)*	
C12	−0.17056 (10)	0.36132 (15)	0.48686 (6)	0.0242 (2)	
H12	−0.1783 (13)	0.481 (2)	0.4670 (9)	0.038 (4)*	
C13	−0.06699 (10)	0.31181 (14)	0.53857 (6)	0.0222 (2)	
H13	−0.0034 (14)	0.387 (2)	0.5534 (9)	0.037 (4)*	
C14	−0.05540 (9)	0.14870 (13)	0.56821 (5)	0.0189 (2)	
C15	0.37851 (10)	−0.06745 (14)	0.77938 (6)	0.0240 (2)	
C16	0.48166 (10)	−0.08838 (14)	0.74715 (6)	0.0227 (2)	
C17	0.58900 (10)	−0.12904 (15)	0.79741 (7)	0.0265 (2)	
H17	0.6567 (14)	−0.143 (2)	0.7741 (9)	0.038 (4)*	
C18	0.59215 (11)	−0.15392 (16)	0.87909 (7)	0.0297 (3)	
H18	0.6648 (14)	−0.188 (2)	0.9122 (10)	0.043 (4)*	
C19	0.49073 (12)	−0.13533 (17)	0.91119 (7)	0.0327 (3)	
H19	0.4947 (14)	−0.153 (2)	0.9695 (10)	0.048 (5)*	
C20	0.38352 (12)	−0.09066 (17)	0.86123 (7)	0.0315 (3)	
H20	0.3124 (14)	−0.0724 (19)	0.8831 (9)	0.035 (4)*	
C21	0.56705 (12)	−0.10204 (19)	0.62942 (7)	0.0329 (3)	
H21A	0.6315 (16)	−0.015 (2)	0.6472 (11)	0.048 (4)*	
H21B	0.5396 (15)	−0.094 (2)	0.5726 (11)	0.047 (4)*	
H21C	0.6026 (15)	−0.216 (2)	0.6451 (10)	0.048 (5)*	

### Atomic displacement parameters (Å^2^)

**Table:**

	*U*^11^	*U*^22^	*U*^33^	*U*^12^	*U*^13^	*U*^23^
O1	0.0201 (4)	0.0221 (4)	0.0365 (4)	−0.0035 (3)	−0.0012 (3)	0.0040 (3)
O2	0.0269 (4)	0.0259 (5)	0.0363 (4)	−0.0078 (3)	0.0020 (3)	−0.0011 (3)
O3	0.0257 (4)	0.0362 (5)	0.0235 (4)	0.0020 (3)	0.0038 (3)	0.0023 (3)
N1	0.0191 (4)	0.0260 (5)	0.0305 (4)	0.0014 (4)	0.0001 (3)	0.0068 (4)
C1	0.0180 (5)	0.0207 (5)	0.0221 (4)	−0.0002 (4)	0.0040 (3)	0.0001 (4)
C2	0.0193 (5)	0.0189 (5)	0.0204 (4)	0.0014 (4)	0.0059 (3)	0.0007 (4)
C3	0.0204 (5)	0.0232 (6)	0.0224 (4)	0.0015 (4)	0.0064 (3)	0.0015 (4)
C4	0.0272 (6)	0.0256 (6)	0.0262 (5)	0.0045 (4)	0.0058 (4)	0.0066 (4)
C5	0.0342 (6)	0.0232 (6)	0.0277 (5)	0.0009 (5)	0.0090 (4)	0.0060 (4)
C6	0.0298 (6)	0.0206 (6)	0.0256 (5)	−0.0033 (5)	0.0089 (4)	0.0015 (4)
C7	0.0217 (5)	0.0199 (5)	0.0201 (4)	−0.0002 (4)	0.0075 (3)	−0.0013 (4)
C8	0.0213 (5)	0.0209 (5)	0.0230 (4)	−0.0018 (4)	0.0066 (3)	−0.0025 (4)
C9	0.0194 (5)	0.0204 (5)	0.0204 (4)	−0.0006 (4)	0.0054 (3)	−0.0027 (4)
C10	0.0196 (5)	0.0252 (6)	0.0244 (4)	−0.0006 (4)	0.0034 (4)	−0.0027 (4)
C11	0.0208 (5)	0.0280 (6)	0.0250 (4)	0.0025 (4)	0.0018 (4)	−0.0017 (4)
C12	0.0233 (5)	0.0218 (6)	0.0269 (5)	0.0032 (4)	0.0037 (4)	0.0007 (4)
C13	0.0203 (5)	0.0206 (5)	0.0254 (4)	−0.0011 (4)	0.0038 (4)	−0.0013 (4)
C14	0.0172 (5)	0.0195 (5)	0.0204 (4)	0.0004 (4)	0.0042 (3)	−0.0013 (4)
C15	0.0217 (5)	0.0235 (6)	0.0251 (4)	0.0016 (4)	0.0002 (4)	0.0023 (4)
C16	0.0231 (5)	0.0193 (5)	0.0243 (4)	0.0002 (4)	0.0014 (4)	−0.0006 (4)
C17	0.0207 (5)	0.0243 (6)	0.0323 (5)	0.0000 (4)	−0.0007 (4)	−0.0004 (4)
C18	0.0280 (6)	0.0256 (6)	0.0305 (5)	0.0007 (5)	−0.0069 (4)	−0.0006 (4)
C19	0.0383 (7)	0.0342 (7)	0.0232 (5)	0.0031 (5)	−0.0002 (4)	0.0014 (4)
C20	0.0312 (6)	0.0365 (7)	0.0270 (5)	0.0047 (5)	0.0059 (4)	0.0031 (5)
C21	0.0300 (6)	0.0394 (8)	0.0307 (5)	−0.0020 (6)	0.0094 (5)	−0.0037 (5)

### Geometric parameters (Å, °)

**Table:**

O1—C1	1.2374 (13)	C10—C11	1.3858 (17)
O2—C8	1.2241 (13)	C10—H10	1.038 (13)
O3—C16	1.3647 (13)	C11—C12	1.3886 (16)
O3—C21	1.4222 (15)	C11—H11	0.978 (14)
N1—C3	1.3600 (15)	C12—C13	1.3884 (14)
N1—C15	1.4238 (13)	C12—H12	1.009 (16)
N1—H1	0.932 (19)	C13—C14	1.3934 (15)
C1—C2	1.4608 (15)	C13—H13	0.937 (16)
C1—C14	1.4892 (14)	C15—C20	1.3887 (16)
C2—C7	1.4128 (15)	C15—C16	1.3996 (16)
C2—C3	1.4293 (14)	C16—C17	1.3912 (15)
C3—C4	1.4122 (16)	C17—C18	1.3910 (16)
C4—C5	1.3726 (17)	C17—H17	0.940 (16)
C4—H4	1.014 (14)	C18—C19	1.3782 (19)
C5—C6	1.3961 (16)	C18—H18	0.948 (16)
C5—H5	1.051 (17)	C19—C20	1.3956 (17)
C6—C7	1.3851 (16)	C19—H19	0.990 (17)
C6—H6	0.983 (15)	C20—H20	0.966 (16)
C7—C8	1.4933 (14)	C21—H21A	1.016 (18)
C8—C9	1.4779 (16)	C21—H21B	0.955 (18)
C9—C14	1.3998 (14)	C21—H21C	1.013 (18)
C9—C10	1.3998 (14)		
			
C16—O3—C21	117.52 (9)	C10—C11—H11	121.5 (8)
C3—N1—C15	124.05 (10)	C12—C11—H11	118.1 (8)
C3—N1—H1	113.9 (12)	C13—C12—C11	120.03 (11)
C15—N1—H1	120.7 (12)	C13—C12—H12	119.3 (9)
O1—C1—C2	122.81 (9)	C11—C12—H12	120.6 (9)
O1—C1—C14	118.80 (10)	C12—C13—C14	120.30 (10)
C2—C1—C14	118.38 (9)	C12—C13—H13	120.7 (10)
C7—C2—C3	118.67 (9)	C14—C13—H13	119.0 (10)
C7—C2—C1	120.35 (9)	C13—C14—C9	119.59 (9)
C3—C2—C1	120.99 (9)	C13—C14—C1	118.73 (9)
N1—C3—C4	120.49 (10)	C9—C14—C1	121.67 (9)
N1—C3—C2	121.15 (10)	C20—C15—C16	119.71 (10)
C4—C3—C2	118.35 (10)	C20—C15—N1	120.68 (11)
C5—C4—C3	121.24 (10)	C16—C15—N1	119.61 (9)
C5—C4—H4	122.7 (8)	O3—C16—C17	124.53 (11)
C3—C4—H4	116.1 (8)	O3—C16—C15	115.56 (9)
C4—C5—C6	121.01 (11)	C17—C16—C15	119.91 (10)
C4—C5—H5	120.9 (9)	C18—C17—C16	119.63 (11)
C6—C5—H5	118.0 (9)	C18—C17—H17	122.3 (9)
C7—C6—C5	119.11 (11)	C16—C17—H17	118.0 (9)
C7—C6—H6	121.3 (9)	C19—C18—C17	120.82 (10)
C5—C6—H6	119.6 (9)	C19—C18—H18	120.3 (10)
C6—C7—C2	121.56 (9)	C17—C18—H18	118.9 (10)
C6—C7—C8	117.08 (10)	C18—C19—C20	119.67 (11)
C2—C7—C8	121.36 (9)	C18—C19—H19	119.7 (10)
O2—C8—C9	121.19 (10)	C20—C19—H19	120.6 (10)
O2—C8—C7	120.98 (10)	C15—C20—C19	120.24 (12)
C9—C8—C7	117.83 (9)	C15—C20—H20	119.0 (9)
C14—C9—C10	119.81 (10)	C19—C20—H20	120.7 (9)
C14—C9—C8	120.32 (9)	O3—C21—H21A	108.9 (10)
C10—C9—C8	119.87 (10)	O3—C21—H21B	106.8 (10)
C11—C10—C9	119.89 (10)	H21A—C21—H21B	109.4 (15)
C11—C10—H10	122.2 (7)	O3—C21—H21C	112.5 (10)
C9—C10—H10	117.8 (7)	H21A—C21—H21C	107.9 (13)
C10—C11—C12	120.35 (10)	H21B—C21—H21C	111.3 (14)
			
O1—C1—C2—C7	−179.01 (10)	C8—C9—C10—C11	179.05 (9)
C14—C1—C2—C7	0.30 (14)	C9—C10—C11—C12	−0.04 (16)
O1—C1—C2—C3	1.50 (16)	C10—C11—C12—C13	1.45 (17)
C14—C1—C2—C3	−179.20 (9)	C11—C12—C13—C14	−1.36 (16)
C15—N1—C3—C4	−0.72 (17)	C12—C13—C14—C9	−0.14 (16)
C15—N1—C3—C2	−179.32 (10)	C12—C13—C14—C1	178.47 (9)
C7—C2—C3—N1	−179.95 (10)	C10—C9—C14—C13	1.55 (15)
C1—C2—C3—N1	−0.44 (16)	C8—C9—C14—C13	−178.96 (9)
C7—C2—C3—C4	1.43 (15)	C10—C9—C14—C1	−177.02 (9)
C1—C2—C3—C4	−179.07 (9)	C8—C9—C14—C1	2.46 (15)
N1—C3—C4—C5	179.81 (11)	O1—C1—C14—C13	−1.90 (15)
C2—C3—C4—C5	−1.55 (16)	C2—C1—C14—C13	178.77 (9)
C3—C4—C5—C6	−0.36 (18)	O1—C1—C14—C9	176.69 (10)
C4—C5—C6—C7	2.38 (17)	C2—C1—C14—C9	−2.64 (15)
C5—C6—C7—C2	−2.49 (16)	C3—N1—C15—C20	−71.76 (16)
C5—C6—C7—C8	176.51 (9)	C3—N1—C15—C16	108.85 (13)
C3—C2—C7—C6	0.58 (15)	C21—O3—C16—C17	6.37 (17)
C1—C2—C7—C6	−178.92 (9)	C21—O3—C16—C15	−173.84 (11)
C3—C2—C7—C8	−178.37 (9)	C20—C15—C16—O3	178.95 (11)
C1—C2—C7—C8	2.13 (15)	N1—C15—C16—O3	−1.66 (16)
C6—C7—C8—O2	−1.10 (15)	C20—C15—C16—C17	−1.25 (18)
C2—C7—C8—O2	177.90 (10)	N1—C15—C16—C17	178.14 (11)
C6—C7—C8—C9	178.70 (9)	O3—C16—C17—C18	−178.15 (11)
C2—C7—C8—C9	−2.30 (14)	C15—C16—C17—C18	2.07 (18)
O2—C8—C9—C14	179.78 (10)	C16—C17—C18—C19	−1.38 (19)
C7—C8—C9—C14	−0.03 (14)	C17—C18—C19—C20	−0.1 (2)
O2—C8—C9—C10	−0.74 (15)	C16—C15—C20—C19	−0.27 (19)
C7—C8—C9—C10	179.46 (9)	N1—C15—C20—C19	−179.65 (12)
C14—C9—C10—C11	−1.46 (16)	C18—C19—C20—C15	1.0 (2)

### Hydrogen-bond geometry (Å, °)

**Table:**

*D*—H···*A*	*D*—H	H···*A*	*D*···*A*	*D*—H···*A*
N1—H1···O1	0.932 (19)	1.886 (18)	2.6279 (13)	135.0 (17)
